# Validation of thoracolumbar injury classification and Severity Score in the management of acute and subacute Osteoporotic vertebral compression fractures – A pilot study and a suggested modification

**DOI:** 10.1016/j.inpm.2024.100438

**Published:** 2024-09-10

**Authors:** Jatinder S. Gill, Martina Stippler, Qing Ruan, Nasir Hussain, Andrew P. White, Vwaire Oruhurhu, Obaid Malik, Thomas Simopoulos, Ivan Urits, Ryan S. D'Souza, Sanjeet Narang, Joshua A. Hirsch

**Affiliations:** a Department of Anesthesiology, Beth Israel Deaconess Medical Center, Harvard Medical School, Boston, MA, USA; b Department of Neurosurgery, Beth Israel Deaconess Medical Center, Harvard Medical School, Boston, MA, USA; c Department of Anesthesiology, The Ohio State University, Wexner Medical Center, Columbus, OH, USA; d Department of Orthopedics, Beth Israel Deaconess Medical Center, Harvard Medical School, Boston, MA, USA; e Department of Anesthesia, Division of Pain Medicine, University of Pittsburgh Medical Center, Susquehanna, PA, USA; f Renner Pain and Spine, Richardson, TX, USA; g Southcoast Health, Pain Management, Wareham, MA, USA; h Department of Anesthesiology and Perioperative Medicine, Mayo Clinic, Rochester, MN, USA; i Department of Radiology, Massachusetts General Hospital, Harvard Medical School, Boston, MA, USA

## Abstract

**Objective:**

To retrospectively assess the Thoracolumbar Injury Classification and Severity Score (TLICS) in patients with osteoporotic vertebral compression fractures (OVCF) and compare the treatment given with that predicted by the TLICS score.

**Methods:**

All medical records of patients presenting from January 2014 to November 2017 for acute atraumatic or low impact OVCF were screened, and eligible patients were retrospectively reviewed. The TLICS score was determined based upon magnetic resonance imaging (MRI) findings and clinical records. Clinical records (including pain score data), imaging data, operative procedures, and stability of neurological examination were tracked over three months for each patient.

**Results:**

Of the 56 patients included, 36 patients had a TLICS score of 1, 18 had a TLICS score of 2, and two had a TLICS score of 4. Only one patient with a TLICS score of 4 underwent surgical stabilization, while the rest of the cohort was managed non-operatively, with or without kyphoplasty. TLICS score 1 corresponded to simple compression and TLICS score 2 corresponded to burst morphology with retropulsion and without neurological deficits. Of the patients with a TLICS score of 1 and 2 who underwent kyphoplasty, there was a statistically significant improvement in pain scores in both groups; however no significant difference was observed, between each TLICS score (i.e., 1 or 2). None of the patients developed instability or neurological decline.

**Conclusion:**

TLICS score correctly predicted operative versus non-operative management in all patients with OVCF. TLICS may be used in making management decisions, and in the triage of these patients for operative versus non-operative evaluations. Our study suggests that patients with TLICS score of 4 or higher require surgical evaluation, while those with TLICS of 1 or 2 are likely to have satisfactory non-surgical management with augmentation or conservative care. In general, patients with OVCF typically present with low TLICS score. Kyphoplasty appears to be similarly beneficial in patients with a TLICS score of 1 or a TLICS score of 2. A modification of the TLICS score by adding TLICS Zero to include uncompressed OVCF with edema is suggested. The limitations of this study include a small size; a larger study is needed to confirm these findings.

## 1 Introduction

Osteoporosis is a common risk factor for atraumatic or low-energy vertebral compression fractures, also known as fragility fractures. Osteoporosis and low bone mass affect nearly one in two US adults over the age of 50 [1]. As the population ages, disease burden from osteoporosis-related fractures is likely to increase. Overall age and sex adjusted incidence of compression fractures is 117 per 100,000. Up to 25 % of women over the age of 50 have sustained a vertebral compression fracture [2]. By 2025, over 3 million osteoporosis-related fractures are expected, which will cost over $25 billion US dollars in direct costs, and $209 billion US dollars in cumulative costs [3].

Osteoporotic vertebral compression fractures (OVCF) can cause significant pain and can inhibit mobility, impacting activities of daily living. Inhibition of mobility is associated with increased risks for thrombo-embolism, pneumonia, and decubitus ulcers. For this reason, OVCF that come to clinical attention are associated with an eight-fold increase in mortality, which is similar to hip fractures in the elderly [4]. The majority of OVCF are stable and do not require operative fixation. Distinguishing the unstable fractures from the stable fractures is important given the significant consequence of missing the opportunity to stabilize the most severe injuries. This distinguishment would be best supported by an easy-to-use method to guide a surgical versus non-surgical referral. That distinction is critical, given the diversity of medical specialties managing these patients, and given the increasing number of OVCF presenting to the outpatient clinic or the emergency room. Current systems of classification proposed for these fractures either do not directly assess operative versus non-operative management and neurologically stability, or alternately, are too complex for a non-spine-surgical physician to determine neurological stability and need for surgical referral [[5], [6], [7], [8], [9], [10], [11]]. The lack of a true standardized method of determining neurological stability and making an operative versus non-operative triage decision leads to ambiguity in the treatment of OVCF, possible over-treatment, possible under-treatment, and suboptimal medical resource utilization.

**Fig. 1:**
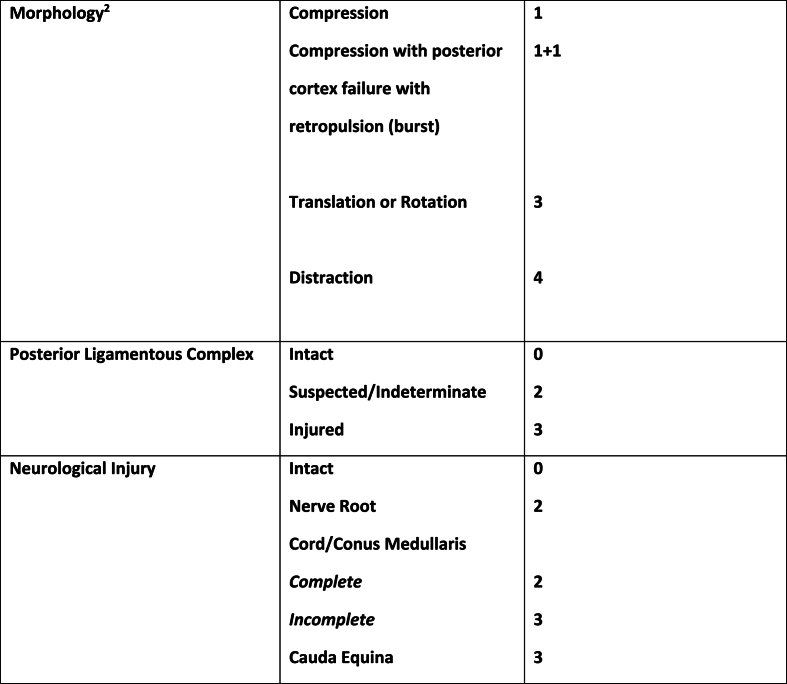
(Created from Vaccaro et al., “A new classification of Thoracolumbar Injuries” Spine. Volume 30)^1^ ^1^A score of 3 or less suggests non-operative injury, score of 5 or more surgical intervention may be considered. A score of 4 might be handled surgically or conservatively. Other clinical qualifiers may allow an otherwise non-surgical patient to become surgical and vice-versa. ^2^In case of multiple morphologies, only one morphology is scored, whichever is highest.

A comprehensive classification system to guide management of OVCF that is succinct, has enough detail and utility, and is reproducible with good inter- and intra-rater agreement has been difficult to create, partly due to the complexity of spinal anatomy and mechanisms of injury, as well as widely differing opinions regarding treatment strategies. The Thoracolumbar Injury Classification and Severity Scale (TLICS) was conceptualized in 2005 based on a survey given to the Spine Trauma Study Group, which consists of worldwide experts in the field of spinal trauma. The TLICS score has a high inter-observer reproducibility. It has been validated and used for guiding the management of traumatic and other high energy vertebral fractures [12] (Fig. 1). The validity of this tool in the management of atraumatic or low-impact OVCFs, however, is not well established. Here we report the TLICS score in a cohort of patients with fragility fractures and assess agreement between the TLICS-predicted treatment and the actual treatment rendered. We also report the outcomes of kyphoplasty in patients with TLICS 1 and TLICS 2 injuries. This is relevant since posterior wall retropulsion (TLICS 2), is considered a kyphoplasty risk factor (due to cement migration into canal or exacerbated retropulsion) and often leads to consideration of surgical stabilization, or avoidance of intervention [10,13]. As such, these TLICS 2 fractures may represent the type of threshold injury that could potentially be undertreated (no kyphoplasty when indicated) or overtreated (strict bed rest, surgery).

## 2 Methods

We conducted a single-institution retrospective cross-sectional study approved by the institutional review board at Beth Israel Deaconess Medical Center. Patients with vertebral compression fractures seen in our pain clinic were identified using an internal database.

### 2.1 Patient sample

We screened records from January 2014 to November 2017 for atraumatic or low impact OVCF. To be eligible for the study, the following inclusion criteria were considered [1]: patients above the age of 60 [2]; acute to subacute thoracolumbar vertebral compression fracture reported on magnetic resonace imaging (MRI) with an elevated short-TI inversion recovery (STIR) signal with no more than mild trauma such as body height fall [3]; documented history and neurological examination that allows for the TLICS score to be accurately calculated. Patients also needed to have a history and physical examination at no less than three months after the initial presentation as well as operative records, if any, in the intervening time.

### 2.2 Data collection

For all eligible patients, the medical records were reviewed, with a specific focus on demographics, clinical notes, radiologic data, pain scores, neurological symptoms, and any procedural notes. Outcomes were collected and stored in a secure encrypted file. Data was collected for the following variables: age; sex; pre-treatment numeric rating scale (NRS) pain score; follow-up NRS pain score; presence of vertebral body height loss on imaging; requirement for surgical intervention; need for external orthosis or brace; and method of OVCF management.

In addition to these variables, the TLICS score was calculated for all patients. The following criterion are included: i) injury morphology from MRI image review as well as radiologist impression (compression, burst, rotation/translation, or distraction); ii) neurologic status (intact, nerve root, incomplete spinal cord or conus medullaris injury, or cauda equina syndrome); and iii) posterior ligamentous complex integrity from image review as well as radiologist impression (intact, suspected/indeterminate, or disrupted) (Fig. 1).

### 2.3 Outcomes assessed

Several outcomes were assessed by our review. Specifically, we primarily sought to quantify the TLICS score amongst patients with OVCFs and their subsequent management. We secondarily sought to quantify the i) pre-intervention mean NRS pain scores, and ii) 3-month mean NRS pain scores. Finally, we also sought to quantify the number of patients with a 50 % improvement in NRS post-management and incidence of post-management loss of vertebral body height.

### 2.4 Outcome measurement

Age was quantified in years and presented as a mean. Gender was presented as proportion of patients who were female. NRS pain scale data was presented as a numeric scale of 0–10, with 10 representing the worst pain imaginable. Data on management OVCF management was presented as an incidence and percent frequency. Data on patients with greater than 50 % NRS pain score improvement and loss of vertebral body height was also presented as incidence form with percent frequency.

### 2.5 Statistical analysis

All statistical analyses in were performed using GraphPad Prism (GraphPad Software, Inc, San Diego, Calif). For the calculation of continuous variables, the paired and unpaired Student t-test was used, and mean data was presented. The ANOVA was used for comparison between multiple groups. For categorical variables, the Fisher's exact test was used and p < 0.05 was defined as the point of statistical significance throughout.

## 3 Results

A total of 105 patients were identified, but only 56 met the inclusion criteria. The rest were excluded due to a lack of complete data up until the 3-month follow-up. Of the 56 patients, 36 had a TLICS score of 1 point; 18 had a score of 2 points; and two had a score of 4 points. There were no patients with TLICS score above 4. Regarding management: 47 patients underwent kyphoplasty, 9 patients were treated conservatively, and 1 kyphoplasty patient underwent subsequent surgical spine stabilization for worsening pain. (Table 1).

**Table 1:** Baseline demographics of patients based on TLICS score alone.

	TLICS 1 (n = 36)	TLICS 2 (n = 18)	TLICS 4 (n = 2)
Mean age	72.7	74.8	68
Fracture site L:T	1:1.9	1:1.3	1:1
Multi-level fractures (n)	2	2	0
Initial mean NRS	7.4	7.9	7.5
F/U mean NRS	4.6	4.2	5.5
>50 % NRS improvement (%)	48.4	55.6	0
Kyphoplasty (n)	30	16	1
Surgery (n)	0	0	1
Brace (n)	11	5	2

Abbreviations: TLICS, Thoracolumbar Injury Classification and Severity Score; F, female; M, male; L, lumbar; T, thoracic; NRS, visual analog scale; n, total patients.

### 3.1 Outcomes in patients managed with kyphoplasty (Table 2, Table 3)

For those patients that underwent kyphoplasty, the mean age of patients with a TLICS score of 1 and 2 were 72.5 and 75.6 years, respectively. The proportion of females with a TLICS score of 1 and 2 were 83 % (25 of 30 patients) and 87 % (14 of 16 patients), respectively. One patient had a TLICS score of 4 and underwent subsequent surgical stabilization for uncontrolled severe pain.

The mean NRS pain scores in patients undergoing kyphoplasty with a TLICS score of 1 and 2 were 8.2 and 8.1 respectively. Similarly, mean NRS scores were 3.9 and 4.2, respectively, at a post procedure follow up visit. Eight patients with a TLICS score of 1 had follow-up imaging within three months, which revealed a single patient with subsequent worsening of the initial vertebral height loss. In contrast, five patients with a TLICS score of 2 had follow-up imaging within three months, with none revealing further height loss. Surgical stabilization was not performed in any patient with a TLICS score of 1 or 2. Braces were prescribed for nine out of 30 patients (30 %) and three of 16 patients (19 %) in patients with a TLICS score of 1 and 2, respectively (Table 2). None of the patients experienced neurological decline. There were no patients in either TLICS 1 or TLICS 2 with cement leakage into the canal.

**Table 2:** – Characteristics and outcomes of patients based on intervention received and comparison of TLICS 1 and TLICS 2 outcomes.

	TLICS 1 with Kyphoplasty (30)	TLICS 2 with Kyphoplasty [16]	No Kyphoplasty [9]
Mean Age (years)	72.5	75.6	64
Sex F:M	25:5	14:2	5:4
Mean baseline NRS (cm)	8.2	8.1	4.2
Follow-up NRS	3.9	4.2	3.5
Patients with >50 % improvement in NRS (n)	13	9	0
Patients with height loss seen on imaging (n)	1/8	0/5	0/6
Surgery required (n)	0	0	0
Brace used (n)	9	3	5

Abbreviations: TLICS, Thoracolumbar Injury Classification and Severity Score; F, female; M, male; NRS, visual analog scale; n, total patients; cm, centimeters.

**Table 3:** – Baseline and follow-up NRS pain score based on intervention received. Data represented as a mean with 95 % confidence interval (95 % CI). For the surgical group, a mean.

	Pre-treatment NRS mean, 95 % CI	Post treatment NRS mean (95 % CI)	p-value
Kyphoplasty	8.2 (7.7–8.8)	4.5 (3.9–5.2)	<0.0001
Conservative Management	4.2 (1.8–6.6)	3.5 (1.5–5.6)	0.41
Surgery	8	6	n/a
p value	<0.0001	0.33	n/a

Abbreviations:; NRS, visual analog scale; CI, confidence interval.

### 3.2 Outcomes in patients managed conservatively (table 2)

Nine patients in total were managed conservatively (without kyphoplasty or surgery). Six of them had a TLICS score of 1; two had a TLICS score of 2; and one had a TLICS score of 4 and was deemed stable for non-operative management via spine surgical evaluation. The mean NRS pain score at the time of presentation and at a follow-up were 4.2 and 3.5 respectively. None of the patients required spine stabilizing surgery. Six out of nine patients during follow-up had subsequent imaging revealing no further vertebral height loss within three months, and five out of nine patients were prescribed braces. For the one patient with a TLICS score of 4, recovery was obtained without neurological deterioration.

### 3.3 Pain scores with different management strategies (Table 2, Table 3)

Pre-treatment baseline NRS pain scores between patients who underwent kyphoplasty, conservative non-interventional management and surgery were demonstrated to be significantly different, with the kyphoplasty group exhibiting markedly higher pain scores {8.2 (7.7–8.8) vs 4.2 (1.8–6.6) p < 0.0001}. In contrast, post treatment NRS pain scores between groups revealed no significant difference. Only patients that underwent kyphoplasty demonstrated significant pre- and post-treatment differences on NRS pain scores (8.2 versus 4.5, p < 0.0001). When comparing patients with a TLICS score of 1 to those with a score of 2, neither group demonstrated pre- or post-treatment differences in NRS pain scores. Further, no difference was seen in the number of subjects with 50 % improvement in NRS post-management, or incidence of post-management loss of vertebral height.

## 4 Discussion

This study analyses baseline TLICS score in a cohort of fragility fractures and assesses agreement between the predicted treatment pathway and the actual pathway followed. The results of this retrospective review suggests that most OVCF patients present with a low TLICS score (54 of 56 patients scored TLICS of 1 or 2). Further, the majority of these patients can be treated with non-operative care, which is in agreement with their predicted pre-treatment TLICS score. As such, TLICS would have accurately guided this care pathway if instituted as a prospective tool to guide care.

Statistically significant pain reductions were seen in OVCF patients with low TLICS scores (TLICS 1 and 2) who underwent kyphoplasty. Significant pain reduction was not seen, however, in similar patients who underwent conservative management only. For those patients with a low TLICS score, there was low rate of progression of radiographic compression and none of the patients developed mechanical instability or neurological decline.

This study establishes the utility of the TLICS score in the management of low-energy “fragility” or Osteoporotic Vertebral Compression Fractures (OVCF) especially for fractures with TLICS score of 1 and 2. Prior studies have established the utility of TLICS score in more severe, or high-energy, thoracolumbar spine trauma, which are less frequent in the general population [14]. Classification of vertebral fractures is important for standardizing communication, conducting research, prognostication, and guiding treatment. Genant classified vertebral fractures into three morphologic categories, wedge, biconcave, and crush, with each category having four grades, normal (Grade 0), mild deformity (Grade 1), moderate deformity (Grade 2), and severe deformity (Grade 3) [5]. Whereas this system is very useful for detecting and describing fractures, it unfortunately provides little guidance regarding the clinical management or neurological stability.

In another attempt to develop a predictive system, Kanchiku et al. evaluated signal characteristics on MRI to prognosticate the outcomes in OVCF [7]. The authors demonstrated higher sensitivity of MRI to X-rays in detecting fractures. Further, they classified the area of T1 weighted intensity change in vertebral body to superior, inferior, anterior, posterior, central, and total. The authors found that fractures with posterior or total types of signal changes were at high risk for intraspinal protrusion. The authors suggested that patients with such morphology should be treated with greater precautions and bed rest, as these were thought to be more unstable. It is challenging, however, as discussed by the authors, to determine operative indication using this classification [7]. Currently, vertebral body signal changes are not clinically used to determine stability.

In another study evaluating the morphology of fractures for prognostication, Sugita et al. assessed the geometry of fractures and the correlation with achieving fusion versus formation of non-healing clefts and progressive collapse [6]. The authors demonstrated that patients with anterior wall morphology defined as swelled-front-type, bow-shaped-type, and projecting-type had a poor prognosis, with late collapse and vacuum clefts; whereas concave-type and dented-type had a good prognosis. In another similar MRI study, Takahashi et al. examined the MRI signal change as diffuse or confined, and high or low. It was found that diffuse low signal change was associated with lower vertebral height, and high signal change was associated with angular motion [11]. Both of these classifications do not assess operative versus non-operative pathway for OVCF.

The most extensive work in determining the need for operative fixation has been done by Schnake et al. and the Spine Section of German Society for Orthopedics and Trauma [[8], [9], [10]]. Their osteoporotic fracture (OF) classification has five categories. OF1 is edema without compression, OF2 is one endplate compressed, OF3 is one end plate fractured with significant posterior wall involvement (>1/5th), OF4 is both end plates fractured with or without posterior wall involvement, OF5 is tension band failure with distraction, translation, or rotation. In addition to these, other factors such as bone density, pain level, co-morbidities, neurological deficits are also considered. Non-operative management either conservative or with cement augmentation is recommended for OF1 and OF2. Operative fixation is recommended for some patients with OF3, and for all patients OF4 and OF5. Using their criteria in 707 patients, they identified 144 fractures, and operative fixation was performed in 30 % of patients. Final choice correlated with the OF score in 85 % of these patients. While this extensive work does help define operative versus non-operative management in OVCF and is of great relevance to a spine surgeon, the overall rate of operative fixation needed in 30 % of OVCF is very high when compared to the U.S. incidence where fewer than 5 % of patients with OVCF are reported to need operative fixation [15]. The rate of mechanical failure of fusion and complications of fusion in OVCF patients has been reported to be as high as 64 % and 70 % respectively [16,17]. Additionally, OF classification is sufficiently complex and cannot be easily adopted by multiple specialties for daily use to determine neurological stability and need for surgical referral.

None of the currently proposed systems of classification for OVCF adequately serve the i) need of ease of use by multiple non-surgical specialties and ii) the triage decision of operative versus non-operative referral by a non-surgical provider. This prompts the need for an additional tool, which is simple to use, reproducible, and comments on the neurological status of patients. The TLICS is one such system that incorporates clinical presentation, components of fracture morphology, as well as assessment of posterior ligamentous complex (PLC) injury. In the absence of an MRI, imputation about PLC injury can also be made from radiographic morphology such as splaying of the spinous processes, diastasis of the facet joint, and widening of the interpedicular distance [12]. The TLICS score has high inter-rater and intra-rater agreement as well good predictability agreement with clinical course followed. The TLICS scoring system is proposed and currently used for high-energy thoracolumbar trauma. The TLICS system can also be used, however, in OVCF patients who usually only have minor trauma such as body height fall and may at times be atraumatic. These can be considered to lie on one end of the spectrum of spinal trauma. The differences between non-osteoporotic and osteoporotic fractures are as follows: a) Osteoporotic fractures are less likely to cause ligamentous and tension band rupture due to their low-trauma nature. b) Osteoporotic fractures are more likely to lead to further collapse and delayed healing because of low bone density. c) Those with osteoporotic fractures are generally less suitable candidates for surgery due to poor bone quality and multiple comorbidities, which increase surgical risks in the elderly [16,17]. OVCF presenting with low TLICS score therefore are more likely to be triaged to non-operative arm as they are poor surgical candidates. The rate of further height loss cannot be predicted, but at some point, these patients may become surgical candidates if they were to develop neurological symptoms or have progressive kyphotic deformity [18]. For patients with grade 4 or higher, surgery would be a consideration by TLICS classification, all of these patients must receive surgical evaluation. In our cohort, the TLICS score was mostly low (54/56 patients) at TLICS 1 and 2, and the treatment pathway was successfully predicted by the TLICS score in all of the patients in our study. This suggests that even burst morphology (TLICS 2), which is not uncommon among OVCF, can be safely managed without need for surgical stabilization. One of the limitations of the TLICS score, however, is the lack of consideration of the degree of posterior wall retropulsion. Many TLICS 2 fractures may be conservatively managed, but severe retropulsion could be indicative of instability and would likely be associated with neurological findings that would likely increase the TLICS score to 4 or higher.

The one important difference between OVCF and fractures related to major trauma is that osteoporotic fractures may present with just edema, and no height loss, but subsequently compress. The TLICS does not address this situation and it is suggested that for OVCF the TLICS score may be modified such that uncompressed fractures may be assigned a TLICS score of zero.

The patients who underwent repeat imaging in all three groups revealed a low rate of further height loss, and brace usage was also infrequent in this cohort. Whereas restriction of heavy physical activity is advisable in these patients, inactivity and bed rest can be deleterious, particularly in this elderly age group. Based upon studies by Kanchiku et al. and based on OF classification, greater precaution may be needed in patients with posterior wall signal change or disruption. Based upon our study, similar outcomes for simple compression or burst morphology in terms of pain can be expected in patients treated with kyphoplasty. Rigid braces are poorly tolerated and suffer from lack of compliance. While bracing may improve comfort for some patients with acute fractures, no significant differences have been found between brace and non-brace groups in terms of long-term outcomes or further height loss [19]. The American Academy of Orthopedics is inconclusive regarding the use of bracing [20]. The low number of patients with subsequent imaging prevents us from drawing any conclusions, however, regarding overall further height loss and the utility of a brace.

Our study demonstrated a lack of improvement in pain scores in the conservative group (treated without kyphoplasty and without surgery). It is possible that this conservatively treated group could have had pain relief if they were treated, but it is also possible that the treatment effect would be less in these patients as compared to those with higher pain scores. Other benefits that are considered to accrue from augmentation including arrest of height loss [21], and early mobilization; this may warrant use of kyphoplasty or vertebral body augmentation, even with lower pain scores. The reason no significant height loss was seen in the non-kyphoplasty group may relate to the low numbers of subjects in this group.

The present study comes with several limitations. The total number of patients is low. A larger study is needed to validate the TLICS score in use of OVCF patients. Most patients underwent kyphoplasty, and only one patient underwent operative surgical stabilization. In the future, studies should be conducted in OVCF patients who receive no interventional treatment to further validate the TLICS score in patients in this population. No patients with TLICS score higher than four were seen in this cohort. This may reflect referral bias but also points to the fact that these are low trauma or atraumatic fractures and higher TLICS scores are unlikely to be seen in this cohort. Furthermore, it validates non operative management in this cohort when TLICS score is low. The study may also be underpowered to detect adverse events. Other limitations of retrospective data such as selection bias and attrition also apply.

## 5 Conclusions

To conclude, OVCF patients appear to largely present with a low TLICS score of 1 and 2, and the score appears to correctly predict the clinical management in this group of patients. Thus, the TLICS score can be used to guide operative versus non-operative decisions. Because of the clarity and simplicity of the TLICS, this is especially helpful for the large fraction of triage and referral decisions made by a non-surgical and non-spine specialty physicians. Kyphoplasty appears to provide a meaningful pain reduction that is similar in patients with simple compression or compression with posterior wall retropulsion. Finally, to address uncompressed OVCF with edema, a modification of TLICS score by adding TLICS zero is suggested. The study limitations include small sample size; a larger study is needed to confirm these findings.

## Funding

No funding was received for this work:

## Conflicts of interest

No conflicts of interests to report.

All data is presented in this manuscript; any additional data is available upon reasonable request.

## Declaration of competing interest

The authors declare that they have no known competing financial interests or personal relationships that could have appeared to influence the work reported in this paper.
